# *N*-(4-Chloro­benzyl­idene)-1-naphthyl­amine

**DOI:** 10.1107/S1600536810032332

**Published:** 2010-08-18

**Authors:** Ruitao Zhu, Yuewen Zhang, Yuehong Ren

**Affiliations:** a Department of Chemistry, Taiyuan Normal University, Taiyuan 030031, People’s Republic of China

## Abstract

The title compound, C_17_H_12_ClN, represents a *trans* isomer with respect to the C=N bond; the dihedral angle between the planes of the naphthyl and benzene groups is 66.53 (5)°.

## Related literature

For general background on the properties of Schiff bases, see: Layer (1963 ▶); Chen *et al.* (2008 ▶); May *et al.* (2004 ▶); Weber *et al.* (2007 ▶). For related structures, see: Harada *et al.* (2004 ▶); Tariq *et al.* (2010 ▶).
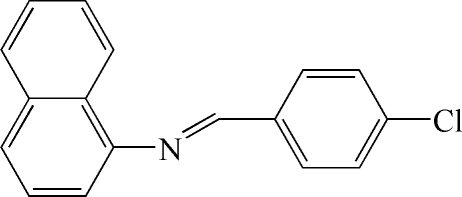


## Experimental

### 

#### Crystal data

C_17_H_12_ClN
*M* *_r_* = 265.73
Monoclinic, 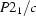

*a* = 12.8416 (13) Å
*b* = 14.8771 (15) Å
*c* = 7.1971 (8) Å
β = 92.857 (1)°
*V* = 1373.3 (2) Å^3^
*Z* = 4
Mo *K*α radiation
μ = 0.26 mm^−1^
*T* = 296 K
0.30 × 0.24 × 0.20 mm

#### Data collection

Bruker APEXII CCD diffractometer
Absorption correction: multi-scan (*SADABS*; Sheldrick, 1996 ▶) *T*_min_ = 0.925, *T*_max_ = 0.949
6607 measured reflections
2421 independent reflections
1489 reflections with *I* > 2σ(*I*)
*R*_int_ = 0.038

#### Refinement

*R*[*F* ^2^ > 2σ(*F* ^2^)] = 0.049
*wR*(*F* ^2^) = 0.127
*S* = 1.03
2421 reflections
172 parameters
H-atom parameters constrained
Δρ_max_ = 0.16 e Å^−3^
Δρ_min_ = −0.22 e Å^−3^

### 

Data collection: *APEX2* (Bruker, 2007 ▶); cell refinement: *SAINT* (Bruker, 2007 ▶); data reduction: *SAINT*; program(s) used to solve structure: *SHELXS97* (Sheldrick, 2008 ▶); program(s) used to refine structure: *SHELXL97* (Sheldrick, 2008 ▶); molecular graphics: *SHELXTL* (Sheldrick, 2008 ▶); software used to prepare material for publication: *SHELXTL*.

## Supplementary Material

Crystal structure: contains datablocks I, global. DOI: 10.1107/S1600536810032332/ya2125sup1.cif

Structure factors: contains datablocks I. DOI: 10.1107/S1600536810032332/ya2125Isup2.hkl

Additional supplementary materials:  crystallographic information; 3D view; checkCIF report

## Figures and Tables

**Fig. 1.**
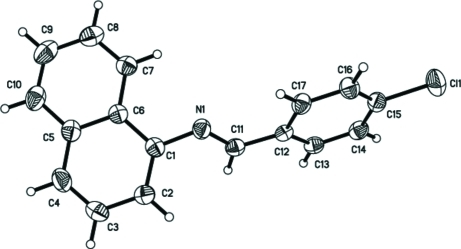
A view of the molecular structure of the title compound; displacement ellipsoids are drawn at the 30% probability level.

## supplementary crystallographic information

### Comment

The Schiff bases have been receiving considerable attention for many years, primarily due to their importance as ligands in metal complexes with special magnetic (Weber *et al.*, 2007), catalytic (Chen *et al.*, 2008) and biological properties (May *et al.*,2004).

As a part of our studies on synthesis and structural peculiarities of Schiff bases derived from naphthylamine and arylaldehydes, we determined the structure of the title compound (Fig. 1). The molecule represents a *trans*-isomer with respect to the C11═N1 bond. The planes of the aromatic systems of the the naphthyl and benzene groups, C1–C10 and C12–C17 respectively, form dihedral angle of 66.53 (5)°.

### Experimental

1-Naphthylamine (0.72 g, 5 mmol) and 4-chlorobenzaldehyde (0.70 g, 5 mmol) were dissolved in ethanol (20 ml). The mixture was refluxed for 6 h, and then cooled to room temperature. The reaction mixture was filtered and the filter cake was recrystallized from ethyl alcohol (yield 80%).Crystals of the title compound suitable for X-ray diffraction were obtained by slow evaporation of an ethanol solution.

### Refinement

H atoms were placed in idealized positions and allowed to ride on their respective parent atoms, with C—H = 0.93 Å and *U*_iso_(H) = 1.2*U*_eq_(C).

### Crystal data

**Table:**

C_17_H_12_ClN	*F*(000) = 552
*M**_r_* = 265.73	*D*_x_ = 1.285 Mg m^−^^3^
Monoclinic, *P*2_1_/*c*	Mo *K*α radiation, λ = 0.71073 Å
Hall symbol: -P 2ybc	Cell parameters from 1396 reflections
*a* = 12.8416 (13) Å	θ = 3.0–21.8°
*b* = 14.8771 (15) Å	µ = 0.26 mm^−^^1^
*c* = 7.1971 (8) Å	*T* = 296 K
β = 92.857 (1)°	Prism, colourless
*V* = 1373.3 (2) Å^3^	0.30 × 0.24 × 0.20 mm
*Z* = 4	

### Data collection

**Table:**

Bruker APEXII CCD diffractometer	2421 independent reflections
Radiation source: fine-focus sealed tube	1489 reflections with *I* > 2σ(*I*)
graphite	*R*_int_ = 0.038
φ and ω scans	θ_max_ = 25.0°, θ_min_ = 2.7°
Absorption correction: multi-scan (*SADABS*; Sheldrick, 1996)	*h* = −15→15
*T*_min_ = 0.925, *T*_max_ = 0.949	*k* = −17→13
6607 measured reflections	*l* = −8→8

### Refinement

**Table:**

Refinement on *F*^2^	Primary atom site location: structure-invariant direct methods
Least-squares matrix: full	Secondary atom site location: difference Fourier map
*R*[*F*^2^ > 2σ(*F*^2^)] = 0.049	Hydrogen site location: inferred from neighbouring sites
*wR*(*F*^2^) = 0.127	H-atom parameters constrained
*S* = 1.03	*w* = 1/[σ^2^(*F*_o_^2^) + (0.0469*P*)^2^ + 0.2941*P*] where *P* = (*F*_o_^2^ + 2*F*_c_^2^)/3
2421 reflections	(Δ/σ)_max_ < 0.001
172 parameters	Δρ_max_ = 0.16 e Å^−^^3^
0 restraints	Δρ_min_ = −0.22 e Å^−^^3^

### Special details

**Table:**

Geometry. All e.s.d.'s (except the e.s.d. in the dihedral angle between two l.s. planes) are estimated using the full covariance matrix. The cell e.s.d.'s are taken into account individually in the estimation of e.s.d.'s in distances, angles and torsion angles; correlations between e.s.d.'s in cell parameters are only used when they are defined by crystal symmetry. An approximate (isotropic) treatment of cell e.s.d.'s is used for estimating e.s.d.'s involving l.s. planes.
Refinement. Refinement of *F*^2^ against ALL reflections. The weighted *R*-factor *wR* and goodness of fit *S* are based on *F*^2^, conventional *R*-factors *R* are based on *F*, with *F* set to zero for negative *F*^2^. The threshold expression of *F*^2^ > σ(*F*^2^) is used only for calculating *R*-factors(gt) *etc*. and is not relevant to the choice of reflections for refinement. *R*-factors based on *F*^2^ are statistically about twice as large as those based on *F*, and *R*-factors based on ALL data will be even larger.

### Fractional atomic coordinates and isotropic or equivalent isotropic displacement parameters (Å^2^)

**Table:**

	*x*	*y*	*z*	*U*_iso_*/*U*_eq_	
Cl1	1.41202 (5)	0.30834 (6)	1.24470 (11)	0.0803 (3)	
N1	0.92436 (15)	0.37176 (15)	0.8844 (3)	0.0512 (6)	
C1	0.81851 (19)	0.36531 (17)	0.8206 (4)	0.0481 (6)	
C2	0.7440 (2)	0.3300 (2)	0.9273 (4)	0.0593 (8)	
H2	0.7634	0.3045	1.0418	0.071*	
C3	0.6383 (2)	0.3315 (2)	0.8669 (4)	0.0733 (9)	
H3	0.5885	0.3071	0.9416	0.088*	
C4	0.6086 (2)	0.3681 (2)	0.7013 (5)	0.0713 (9)	
H4	0.5383	0.3691	0.6635	0.086*	
C5	0.6828 (2)	0.40512 (19)	0.5839 (4)	0.0553 (7)	
C6	0.78968 (18)	0.40408 (17)	0.6440 (3)	0.0456 (6)	
C7	0.8628 (2)	0.44051 (18)	0.5263 (4)	0.0545 (7)	
H7	0.9331	0.4402	0.5642	0.065*	
C8	0.8331 (3)	0.4761 (2)	0.3586 (4)	0.0705 (9)	
H8	0.8830	0.4996	0.2829	0.085*	
C9	0.7281 (3)	0.4776 (2)	0.2989 (4)	0.0786 (10)	
H9	0.7083	0.5022	0.1837	0.094*	
C10	0.6545 (2)	0.4433 (2)	0.4087 (4)	0.0710 (9)	
H10	0.5847	0.4449	0.3679	0.085*	
C11	0.97066 (19)	0.30238 (18)	0.9492 (3)	0.0484 (6)	
H11	0.9351	0.2479	0.9460	0.058*	
C12	1.07745 (18)	0.30453 (17)	1.0286 (3)	0.0422 (6)	
C13	1.13264 (19)	0.22522 (18)	1.0563 (3)	0.0482 (6)	
H13	1.0997	0.1706	1.0306	0.058*	
C14	1.23542 (19)	0.2257 (2)	1.1212 (3)	0.0535 (7)	
H14	1.2720	0.1721	1.1381	0.064*	
C15	1.28284 (18)	0.3068 (2)	1.1604 (3)	0.0506 (7)	
C16	1.22982 (19)	0.3865 (2)	1.1386 (3)	0.0544 (7)	
H16	1.2628	0.4406	1.1686	0.065*	
C17	1.12764 (19)	0.38576 (18)	1.0719 (3)	0.0512 (7)	
H17	1.0917	0.4397	1.0556	0.061*	

### Atomic displacement parameters (Å^2^)

**Table:**

	*U*^11^	*U*^22^	*U*^33^	*U*^12^	*U*^13^	*U*^23^
Cl1	0.0504 (4)	0.1022 (8)	0.0871 (6)	0.0088 (4)	−0.0083 (4)	−0.0073 (5)
N1	0.0495 (12)	0.0453 (14)	0.0580 (14)	−0.0005 (10)	−0.0057 (10)	0.0032 (11)
C1	0.0474 (14)	0.0401 (16)	0.0566 (16)	−0.0019 (12)	0.0021 (12)	−0.0045 (13)
C2	0.0577 (16)	0.064 (2)	0.0564 (17)	−0.0055 (14)	0.0024 (13)	0.0084 (15)
C3	0.0532 (17)	0.087 (3)	0.080 (2)	−0.0136 (16)	0.0116 (15)	0.0087 (19)
C4	0.0469 (16)	0.076 (2)	0.090 (2)	−0.0074 (15)	−0.0090 (16)	0.0031 (19)
C5	0.0555 (16)	0.0449 (17)	0.0643 (18)	−0.0041 (13)	−0.0087 (14)	−0.0053 (14)
C6	0.0491 (14)	0.0347 (15)	0.0527 (16)	−0.0026 (11)	0.0008 (12)	−0.0058 (13)
C7	0.0585 (16)	0.0439 (17)	0.0614 (18)	−0.0009 (13)	0.0067 (13)	−0.0023 (14)
C8	0.089 (2)	0.059 (2)	0.064 (2)	−0.0085 (17)	0.0048 (17)	0.0063 (16)
C9	0.107 (3)	0.063 (2)	0.063 (2)	−0.011 (2)	−0.0192 (19)	0.0083 (17)
C10	0.074 (2)	0.059 (2)	0.077 (2)	−0.0077 (17)	−0.0272 (17)	0.0013 (18)
C11	0.0543 (15)	0.0436 (17)	0.0476 (14)	−0.0036 (13)	0.0042 (12)	0.0021 (13)
C12	0.0498 (13)	0.0404 (16)	0.0366 (13)	−0.0005 (12)	0.0034 (10)	0.0047 (12)
C13	0.0569 (15)	0.0388 (16)	0.0495 (15)	−0.0004 (13)	0.0080 (12)	0.0035 (13)
C14	0.0576 (16)	0.0502 (18)	0.0531 (16)	0.0134 (14)	0.0082 (13)	0.0073 (14)
C15	0.0462 (14)	0.0599 (19)	0.0457 (15)	0.0067 (14)	0.0038 (11)	−0.0010 (14)
C16	0.0536 (15)	0.0518 (19)	0.0574 (17)	−0.0033 (14)	−0.0015 (13)	−0.0066 (14)
C17	0.0561 (15)	0.0435 (17)	0.0534 (16)	0.0048 (13)	−0.0033 (12)	0.0022 (13)

### Geometric parameters (Å, °)

**Table:**

Cl1—C15	1.738 (2)	C8—H8	0.9300
N1—C11	1.268 (3)	C9—C10	1.361 (4)
N1—C1	1.416 (3)	C9—H9	0.9300
C1—C2	1.361 (4)	C10—H10	0.9300
C1—C6	1.427 (3)	C11—C12	1.459 (3)
C2—C3	1.405 (4)	C11—H11	0.9300
C2—H2	0.9300	C12—C13	1.386 (3)
C3—C4	1.348 (4)	C12—C17	1.397 (3)
C3—H3	0.9300	C13—C14	1.378 (3)
C4—C5	1.416 (4)	C13—H13	0.9300
C4—H4	0.9300	C14—C15	1.375 (4)
C5—C10	1.414 (4)	C14—H14	0.9300
C5—C6	1.419 (3)	C15—C16	1.372 (4)
C6—C7	1.404 (3)	C16—C17	1.375 (3)
C7—C8	1.355 (4)	C16—H16	0.9300
C7—H7	0.9300	C17—H17	0.9300
C8—C9	1.395 (4)		
			
C11—N1—C1	119.3 (2)	C10—C9—H9	119.9
C2—C1—N1	122.2 (2)	C8—C9—H9	119.9
C2—C1—C6	120.0 (2)	C9—C10—C5	120.9 (3)
N1—C1—C6	117.6 (2)	C9—C10—H10	119.5
C1—C2—C3	121.0 (3)	C5—C10—H10	119.5
C1—C2—H2	119.5	N1—C11—C12	122.7 (2)
C3—C2—H2	119.5	N1—C11—H11	118.6
C4—C3—C2	120.5 (3)	C12—C11—H11	118.6
C4—C3—H3	119.8	C13—C12—C17	118.5 (2)
C2—C3—H3	119.8	C13—C12—C11	120.1 (2)
C3—C4—C5	121.0 (3)	C17—C12—C11	121.3 (2)
C3—C4—H4	119.5	C14—C13—C12	121.2 (2)
C5—C4—H4	119.5	C14—C13—H13	119.4
C10—C5—C4	122.5 (3)	C12—C13—H13	119.4
C10—C5—C6	118.5 (3)	C15—C14—C13	118.8 (2)
C4—C5—C6	118.9 (3)	C15—C14—H14	120.6
C7—C6—C5	118.5 (2)	C13—C14—H14	120.6
C7—C6—C1	122.8 (2)	C16—C15—C14	121.5 (2)
C5—C6—C1	118.7 (2)	C16—C15—Cl1	119.2 (2)
C8—C7—C6	121.4 (3)	C14—C15—Cl1	119.2 (2)
C8—C7—H7	119.3	C15—C16—C17	119.5 (3)
C6—C7—H7	119.3	C15—C16—H16	120.2
C7—C8—C9	120.4 (3)	C17—C16—H16	120.2
C7—C8—H8	119.8	C16—C17—C12	120.4 (2)
C9—C8—H8	119.8	C16—C17—H17	119.8
C10—C9—C8	120.2 (3)	C12—C17—H17	119.8
			
C11—N1—C1—C2	52.2 (4)	C6—C7—C8—C9	0.2 (4)
C11—N1—C1—C6	−132.8 (2)	C7—C8—C9—C10	−0.1 (5)
N1—C1—C2—C3	174.4 (3)	C8—C9—C10—C5	−0.4 (5)
C6—C1—C2—C3	−0.4 (4)	C4—C5—C10—C9	−179.5 (3)
C1—C2—C3—C4	0.1 (5)	C6—C5—C10—C9	0.6 (4)
C2—C3—C4—C5	0.5 (5)	C1—N1—C11—C12	−176.0 (2)
C3—C4—C5—C10	179.4 (3)	N1—C11—C12—C13	−164.6 (2)
C3—C4—C5—C6	−0.7 (5)	N1—C11—C12—C17	13.0 (4)
C10—C5—C6—C7	−0.4 (4)	C17—C12—C13—C14	−1.4 (4)
C4—C5—C6—C7	179.7 (3)	C11—C12—C13—C14	176.3 (2)
C10—C5—C6—C1	−179.8 (2)	C12—C13—C14—C15	0.7 (4)
C4—C5—C6—C1	0.3 (4)	C13—C14—C15—C16	0.8 (4)
C2—C1—C6—C7	−179.1 (3)	C13—C14—C15—Cl1	179.32 (19)
N1—C1—C6—C7	5.8 (4)	C14—C15—C16—C17	−1.5 (4)
C2—C1—C6—C5	0.2 (4)	Cl1—C15—C16—C17	180.0 (2)
N1—C1—C6—C5	−174.9 (2)	C15—C16—C17—C12	0.7 (4)
C5—C6—C7—C8	0.0 (4)	C13—C12—C17—C16	0.7 (4)
C1—C6—C7—C8	179.3 (3)	C11—C12—C17—C16	−177.0 (2)
